# Temporal evolution of new T1-weighted hypo-intense lesions and central brain atrophy in patients with a first clinical demyelinating event treated with subcutaneous interferon β-1a

**DOI:** 10.1007/s00415-022-11554-5

**Published:** 2023-02-01

**Authors:** H. Vrenken, M. Battaglini, M. L. de Vos, G. J. Nagtegaal, B. C. A. Teixeira, A. Seitzinger, D. Jack, M. P. Sormani, B. M. J. Uitdehaag, A. Versteeg, G. Comi, L. Kappos, N. De Stefano, F. Barkhof

**Affiliations:** 1 Department of Radiology and Nuclear Medicine, Amsterdam UMC, Location VUmc, Amsterdam, The Netherlands; 2 Department of Medicine, Surgery and Neurosciences, University of Siena, Siena, Italy; 3 Department of Radiology, Federal University of Paraná, Curitiba, Paraná Brazil; 4 Neuroradiology Department, Neurological Institute of Curitiba (INC/CETAC), Curitiba, Paraná Brazil; 5 Global Biostatistics, Merck Healthcare KGaA, Darmstadt, Germany; 6 Global Medical Affairs, Merck Serono Ltd, (an affiliate of Merck KGaA), Feltham, UK; 7 Department of Health Sciences, University of Genoa and Ospedale Policlinico San Martino IRCCS, Genoa, Italy; 8 Department of Neurology, Amsterdam UMC, Location VUmc, Amsterdam, The Netherlands; 9 Università Vita-Salute San Raffaele, Casa di Cura Privata del Policlinico, Milan, Italy; 10 Research Center for Clinical Neuroimmunology and Neuroscience Basel (RC2NB) and Neurology Departments of Head, Spine and Neuromedicine, Biomedical Engineering and Clinical Research, University Hospital, University of Basel, Basel, Switzerland; 11 UCL Institutes of Neurology and Healthcare Engineering, London, UK; 12 Present Address: Department of Radiology and Nuclear Medicine, Biomedical Imaging Group Rotterdam, Erasmus MC-University Medical Center Rotterdam, Rotterdam, The Netherlands

**Keywords:** First clinical demyelinating event, Interferon β-1a, White matter tracts, Lesion evolution, Black-hole lesions, Brain atrophy

## Abstract

**Objective:**

Evaluate the effect of subcutaneous interferon β-1a (sc IFN β-1a) versus placebo on the evolution of T1-weighted MRI lesions and central brain atrophy in in patients with a first clinical demyelinating event (FCDE).

**Methods:**

Post hoc analysis of baseline-to-24 month MRI data from patients with an FCDE who received sc IFN β-1a 44 μg once- (qw) or three-times-weekly (tiw), or placebo, in REFLEX. Patients were grouped according to treatment regimen or conversion to clinically definite MS (CDMS) status. The intensity of new lesions on unenhanced T1-weighted images was classified as T1 iso- or hypo-intense (black holes) and percentage ventricular volume change (PVVC) was assessed throughout the study.

**Results:**

In patients not converting to CDMS, sc IFN β-1a tiw or qw, versus placebo, reduced the overall number of new lesions (*P* < 0.001 and *P* = 0.005) and new T1 iso-intense lesions (*P* < 0.001 and *P* = 0.002) after 24 months; only sc IFN β-1a tiw was associated with fewer T1 hypo-intense lesions versus placebo (*P* < 0.001). PVVC findings in patients treated with sc IFN β-1a suggested pseudo-atrophy that was ~ fivefold greater versus placebo in the first year of treatment (placebo 1.11%; qw 4.28%; tiw 6.76%; *P* < 001); similar findings were apparent for non-converting patients.

**Conclusions:**

In patients with an FCDE, treatment with sc IFN β-1a tiw for 24 months reduced the number of new lesions evolving into black holes.

**Supplementary Information:**

The online version contains supplementary material available at 10.1007/s00415-022-11554-5.

## Introduction

In patients with a first clinical demyelinating event (FCDE), early treatment with interferons (IFN) delays the onset of clinically definite MS (CDMS) [1–5]. In the REFLEX study, treatment of patients with an FCDE with subcutaneous (sc) IFN β-1a significantly improved the secondary endpoints of conventional MRI outcomes, such as white matter (WM) lesion number and volume, compared with placebo [5, 6]. WM lesions represent regions of axonal demyelination; some lesions appear hypo-intense (“black holes”) on unenhanced T1-weighted images, a finding strongly correlated with reduced axonal density [7–9]. Iso-intense lesions may evolve to become hypo-intense, or vice versa upon remyelination [10], with persistent black holes occurring when axonal demyelination and damage are irreversible [10]. These black-hole lesions depict the focal component of degenerative disease processes in MS, while brain atrophy, which starts early in the disease course (i.e., during an FCDE) [11], represents the more widespread component of the degenerative process. Assessing both brain atrophy and the evolution of new lesions into black holes could therefore provide a comprehensive window on degenerative disease processes and the destructiveness of the disease in individual patients. Both measures are accessible from standard MRI and provide a stronger correlation with disability severity than the traditional lesion measures, such as number and volume of new T2-weighted lesions [11, 12].

To advance understanding of how degenerative disease processes unfold early in MS, the evolution of new T1 lesions and central brain atrophy were quantitatively assessed in a large cohort of patients with an FCDE from the REFLEX trial. Specifically, we investigated how these lesions and atrophy responded to treatment with sc IFN β-1a and how they are related to the conversion from an FCDE to CDMS. Central brain atrophy was evaluated based on the fact that the previous studies [13] have already recognized periventricular atrophy as playing a bigger role than peripheral GM atrophy in patients with MS who have 3 years of follow-up.

## Patients and methods

### Patients

In REFLEX (ClinicalTrials.gov identifier: NCT00404352), 517 patients with a FCDE were randomized to early treatment with sc IFN β-1a 44 μg three-times-weekly (tiw; *n* = 171) or once-weekly (qw, *n* = 175), or placebo (*n* = 171) for 24 months [5, 6]. Upon conversion to CDMS, patients were switched to open-label sc IFN β-1a 44 μg tiw.

This post hoc analysis assessed the effects of the two dosing frequencies of sc IFN β-1a 44 μg on the evolution of new T1 lesions and central brain atrophy in patients from the REFLEX trial for whom MRI scans were available at screening and Month 12 (M12), and who had 2 or more scans available after M12 (including a Month 24/treatment termination scan [M24]). Patients in each treatment group were categorized as CDMS converters or non-converters. The total population of patients who converted to CDMS was heterogeneous, containing a mixture of patients who were either treated solely with sc IFN β-1a 44 μg tiw, or who received placebo or sc IFN β-1a 44 μg qw before being switched to sc IFN β-1a 44 μg tiw at the time of conversion (timing of treatment change was, therefore, variable across patients).

To assess the longitudinal effect of treatment over a 24 month period in a homogenous converter population, patients who converted to CDMS during treatment with placebo or sc IFN β-1a 44 μg qw were excluded. The analysis population, therefore, included eligible patients who received placebo or sc IFN β-1a 44 μg qw and did not convert to CDMS, along with patients who received sc IFN β-1a 44 μg tiw and either did or did not convert to CDMS. This meant that all analysed patients who converted to CDMS had been continuously treated with sc IFN β-1a 44 μg tiw throughout the study period. The patient disposition for these post hoc analyses is described in Supplementary Fig. 1.

### Post hoc MRI analyses

All T1-weighted MRI scans acquired between screening and M24 were analysed to determine the evolution of new lesions. Scans acquired at screening, M12 and M24 were used in the central atrophy analyses. T1-weighted MRI images were obtained using a spin-echo sequence and provided 2D images with voxel dimensions of 1 × 1 × 3 mm^3^. Image processing was conducted with tools from the functional MRI brain (FMRIB) software library (FSL; version 5.0.6).

#### Evolution of new lesions: development of black holes

The methodology for assessing the evolution of new lesions as black holes is reported elsewhere [14]. Briefly, the intensity of a new lesion on T1-weighted images prior to gadolinium administration was assessed at each time point, starting at its appearance. Lesion intensities were evaluated visually by experienced raters (supervised by FB [co-author]), and a highly experienced rater inspected the data for outliers and spurious correlations by means of quality control. Lesions were classified as iso-intense in relation to the surrounding WM or hypo-intense (black holes) with respect to neighbouring grey matter (GM), and lesion intensity was then recorded on a standardized form and subsequently digitized in a custom database. It should be noted that lesions with intensities between WM and GM were not considered. Raters were blinded to all patient information including treatment group and conversion status.

Outcomes of interest were new lesion intensity (iso- or hypo-intense) at M24; new lesion evolution (lesions were classified into four categories based on their intensity at first appearance and at M24: iso–iso, iso–hypo, hypo–iso and hypo–hypo-intense, reflecting the extent and development/persistence of demyelination and axonal loss); and the predominant intensity (iso- or hypo-) of new lesions during the study, determined by assessing intensity at the majority of time points (if lesions appeared iso- and hypo-intense at the same number of time points, they were not classified into either category).

#### Central atrophy

Pre-processing of MRI images was performed using the following methodology. First, correction of slice-to-slice intensity variation of the T1-weighted images was performed if the two interleaved sets of slices differed too much in intensity. This was achieved by normalizing the average signal inside the manually edited brain mask created during the primary trial analyses. The brain mask images were obtained by manually modifying the output of the Brain Extraction Tool (BET) algorithm, the guidelines for which are described elsewhere [15]. A maximum variation of 2% between odd and even slice sets was permitted based on heuristic optimization of a small subset of data. In brief, a T1-weighted image was derived from the two image packages (one for the odd and one for the even slices). Then, R_odd_ and R_even_ regressions were obtained, with R_odd_(Z) and R_even_(Z) predicting at Z slice, respectively, the intensity extrapolated from intensity of odd and even slices. Finally, an additive factor for each slice was imposed to derive the “predicted” average intensity: (R_odd_(Z) + R_even_(Z))/2. If the T1-weighted image after correction had an intensity difference between slices lower than 2% we considered the equalization successfully performed. The regression was quadratic in intensity (this choice was made by looking at the intensity at each slice from images used to obtain the within-subject template). To minimize the influence of lesions on atrophy measurements, lesion filling was performed. The lesion masks from T2-weighted images, created in the primary trial analyses, were co-registered onto the corresponding T1-weighted image by way of nearest neighbor interpolation using FSL-FLIRT [16, 17]. Lesion voxels were subsequently replaced by local WM intensities using the lesion-filling tool in FSL, the co-registered lesion masks and the WM segmentation mask, the latter of which was obtained from the T1-weighted image through the FSL-FAST segmentation method in FSL [18, 19].

Ventricular enlargement analysis was then performed. The VIENA method was used to analyse the FSL-SIENA edge displacement maps to quantify the total percentage ventricular volume change (PVVC) [20]. This was achieved using the implementation incorporated in FSL version 5.0.6 as the “-V” option in the FSL-SIENA software.

### Statistical analysis

Tukey’s Hinges were used to calculate the 25th (Q1), 50th (median), and 75th (Q3) percentiles for each variable. Comparisons across all treatment groups were assessed using Kruskal–Wallis tests, and Mann–Whitney *U* tests were used for pairwise comparisons.

For each variable in the evolution of new lesions and central atrophy analyses, comparisons were made for: (1) the three treatment arms within the group of patients who did not convert to CDMS, and (2) patients who converted versus those who did not convert to CDMS within the sc IFN β-1a 44 μg tiw treatment group. For lesion measures, we focused on absolute numbers rather than fractions, as absolute numbers have greater relevance within a clinical setting [14]. All statistical tests were performed using SPSS version 22 (IBM).

### Standard protocol approvals, registrations, and patient consents

This post hoc study used data from the REFLEX trial, which was undertaken in compliance with the Declaration of Helsinki and standards of Good Clinical Practice according to the International Conference on Harmonisation of Technical Requirements for Registration of Pharmaceuticals for Human Use. Before initiation of the trial at each center, the relevant institutional review board or independent ethics committee reviewed and approved the trial protocol, patient information leaflet, informed consent forms, and investigator brochure. All patients provided written informed consent at the screening visit.

### Data availability statement

Any requests for data by qualified scientific and medical researchers for legitimate research purposes will be subject to Merck’s Data Sharing Policy. All requests should be submitted in writing to Merck’s data sharing portal https://www.merckgroup.com/en/research/our-approach-to-research-and-development/healthcare/clinical-trials/commitment-responsible-data-sharing.html. When Merck has a co-research, co-development, or co-marketing or co-promotion agreement, or when the product has been out-licensed, the responsibility for disclosure might be dependent on the agreement between parties. Under these circumstances, Merck will endeavour to gain agreement to share data in response to requests.

## Results

### Evolution of new lesions: development of black holes

After excluding patients who converted to CDMS during treatment with placebo or IFN sc β-1a 44 μg qw (see “Patients”), MRI scans from 314 patients were available for analysis (sc IFN β-1a 44 μg tiw, *n* = 128 [converters to CDMS, *n* = 26; non-converters, *n* = 102]; sc IFN β-1a 44 μg qw, *n* = 105 [all non-converters]; placebo, *n* = 81 [all non-converters]; Supplementary Fig. 1). Patient baseline demographics and MRI characteristics of the groups were generally similar (Table 1).

**Table 1:** Baseline demographics and characteristics of patients with MRI scans included in the evolution of lesions and central atrophy analyses, according to treatment group

Characteristic	Evolution of lesions analysis population			Central atrophy analysis population		
	Placebo (*n* = 81)	sc IFN β-1a 44 μg qw (*n* = 105)	sc IFN β-1a 44 μg tiw (*n* = 128)	Placebo (*n* = 77)	sc IFN β-1a 44 μg qw (*n* = 98)	sc IFN β-1a 44 μg tiw (*n* = 116)
Baseline characteristics						
Age						
Mean (SD)	31.6 (8.1)	31.5 (8.4)	31.5 (8.7)	31.8 (8.2)	31.4 (8.3)	31.3 (8.5)
Median (Q1, Q3)	30.0 (26.0, 37.0)	31.0 (25.0, 38.0)	30.0 (24.0, 37.0)	30.0 (26.0, 37.0)	31.0 (25.0, 38.0)	30.0 (24.0, 36.5)
Female, *n* (%)	53 (65.4)	69 (65.7)	82 (64.1)	52 (67.5)	65 (66.3)	71 (61.2)
Classification of FCDE as monofocal^a^, *n* (%)	46 (56.8)	59 (56.2)	68 (53.1)	43 (55.8)	55 (56.1)	62 (53.4)
Steroid use at FCDE, *n* (%)	56 (69.1)	73 (69.5)	89 (69.5)	53 (68.8)	69 (70.4)	82 (70.7)
EDSS score						
Mean (SD)	1.4 (0.8)	1.5 (0.7)	1.6 (0.8)	1.4 (0.8)	1.6 (0.7)	1.6 (0.8)
Median (Q1, Q3)	1.5 (1.0, 2.0)	1.5 (1.0, 2.0)	1.5 (1.0, 2.0)	1.5 (1.0, 2.0)	1.5 (1.0, 2.0)	1.5 (1.0, 2.00)
MRI characteristics						
T1 Gd+ lesions, *n*	81	105	128	77	98	116
Mean (SD)	0.8 (1.5)	1.1 (2.5)	1.1 (2.1)	0.7 (1.5)	1.1 (2.6)	1.1 (2.0)
Median (Q1, Q3)	0.0 (0.0, 1.0)	0.0 (0.0, 1.0)	0.0 (0.0, 1.0)	0.0 (0.0, 1.0)	0.0 (0.0, 1.0)	0.0 (0.0, 1.0)
Presence of at least 1 T1 Gd + lesion, *n* (%)	28 (34.6)	43 (41.0)	51 (39.8)	25 (32.5)	38 (38.8)	44 (37.9)
T1 Gd + lesion volume, mm^3^						
Mean (SD)	90.7 (209.2)	120.5 (291.3)	140.2 (389.5)	79.6 (194.8)	116.4 (296.7)	110.8 (333.4)
Median (Q1, Q3)	0.0 (0.0, 94.4)	0.0 (0.0, 74.4)	0.0 (0.0, 88.7)	0.00 (0.0, 77.2)	0.0 (0.0, 68.6)	0.0 (0.0, 78.5)
T1 hypo-intense lesions, *n*	81	105	128	77	98	116
Mean (SD)	4.1 (4.6)	5.8 (7.8)	6.5 (7.3)	4.1 (4.7)	6.0 (8.0)	6.8 (7.5)
Median (Q1, Q3)	3.0 (1.0, 5.0)	3.0 (1.0, 7.0)	4.0 (1.0, 9.0)	3.0 (1.0, 5.0)	3.0 (1.0, 7.0)	5.0 (1.0, 10.0)
T1 hypo-intense lesion volume, mm^3^						
Mean (SD)	556.6 (851.9)	675.7 (1229.1)	789.1 (1167.7)	554.9 (858.6)	698.9 (1267.3)	820.4 (1194.9)
Median (Q1, Q3)	172.8 (17.2, 781.1)	168.8 (42.9, 709.5)	307.6 (70.1, 1090.1)	172.8 (17.2, 781.1)	167.4 (42.9, 723.8)	331.3 (70.1, 1120.1)
T2 lesions, *n*	81	105	128	77	98	116
Mean (SD)	16.5 (14.7)	23.1 (21.6)	22.3 (17.1)	16.8 (14.8)	23.1 (21.7)	22.8 (17.6)
Median (Q1, Q3)	13.0 (6.0, 22.0)	17.0 (8.0, 33.0)	19.5 (10.0, 29.0)	13.0 (6.0, 22.0)	17.0 (8.0, 33.0)	20.0 (10.0, 29.0)
≥ 9 T2 lesions^b^, *n* (%)	54 (66.7)	74 (70.5)	100 (78.1)	52 (67.5)	69 (70.4)	91 (78.4)
T2 lesion volume, mm^3^						
Mean (SD)	2766.9 (3519.0)	3182.2 (3931.9)	3201.0 (3267.9)	2776.6 (3570.3)	3014.5 (3790.6)	3193.4 (3334.7)
Median (Q1, Q3)	1344.6 (682.5, 3178.6)	1682.3 (568.5, 3616.3)	22,016 (894.1, 3899.6)	1287.7 (682.5, 3178.6)	1678.4 (543.6, 3596.4)	2190.1 (894.1, 3855.2)
Normalized brain volume, cm^3^, *n*	81	105	127^c^	77	98	116
Mean (SD)	1542.5 (60.0)	1533.9 (60.3)	1532.2 (77.2)	1542.9 (59.7)	1532.9 (61.4)	1532.3 (79.0)
Median (Q1, Q3)	1546.3 (1506.8, 1582.2)	1537.0 (1502.1, 1569.7)	1533.5 (1473.0, 1597.8)	1547.6 (1507.6, 1582.2)	1537.2 (1500.8, 1569.7)	1535.2 (1472.7, 1598.3)

*CDMS* clinically definite multiple sclerosis, *EDSS* expanded disability status scale, *FCDE* first clinical demyelinating event, *Gd* + gadolinium-enhancing, *Q* quartile, *qw* once-weekly, *IFN* interferon, *sc* subcutaneous, *SD* standard deviation, *tiw* three-times-weekly

^a^According to the adjudication committee

^b^No patients had 0 or 1 T2 lesion

^c^One patient missing due to technical errors

#### Black-hole lesion counts in non-converters

**Fig. 1:**
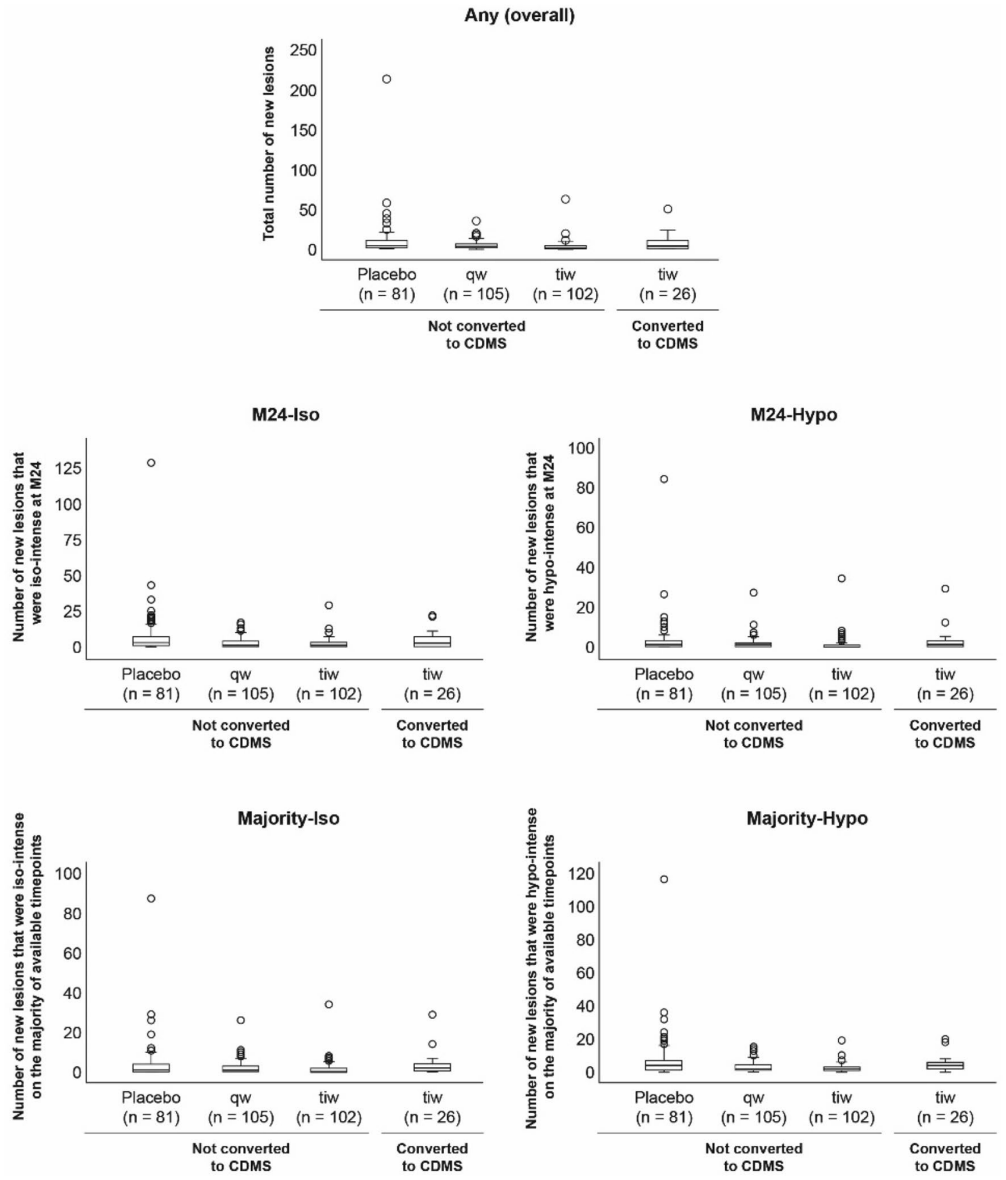
Box and whisker plots of new lesions identified at month 24 in patients with a first clinical demyelinating event and treated with placebo or subcutaneous interferon β-1a (once- or three-times-weekly) and predominant lesion intensities during the study period. Findings are shown according to whether patients converted to clinically definite multiple sclerosis or not. Outcomes reported were defined as follows. Any (overall): cumulative number of new lesions per patient up to and including M24; M24-Iso: number of new lesions that were iso-intense at the M24 time point; M24-Hypo: number of new lesions that were hypo-intense at the M24 time point. The lower panels concern the predominant intensity (iso- or hypo-) of new lesions during the study, determined by intensity at the majority of time points (if lesions appeared iso- and hypo-intense at the same number of time points, they were not classified into either category): Majority-Iso: number of new lesions that were iso-intense on the majority of available time points; Majority-Hypo: number of new lesions that were hypo-intense on the majority of available time points. *CDMS* clinically definite multiple sclerosis, *M* month, *qw* once-weekly, *tiw* three-times-weekly

**Fig. 2:**
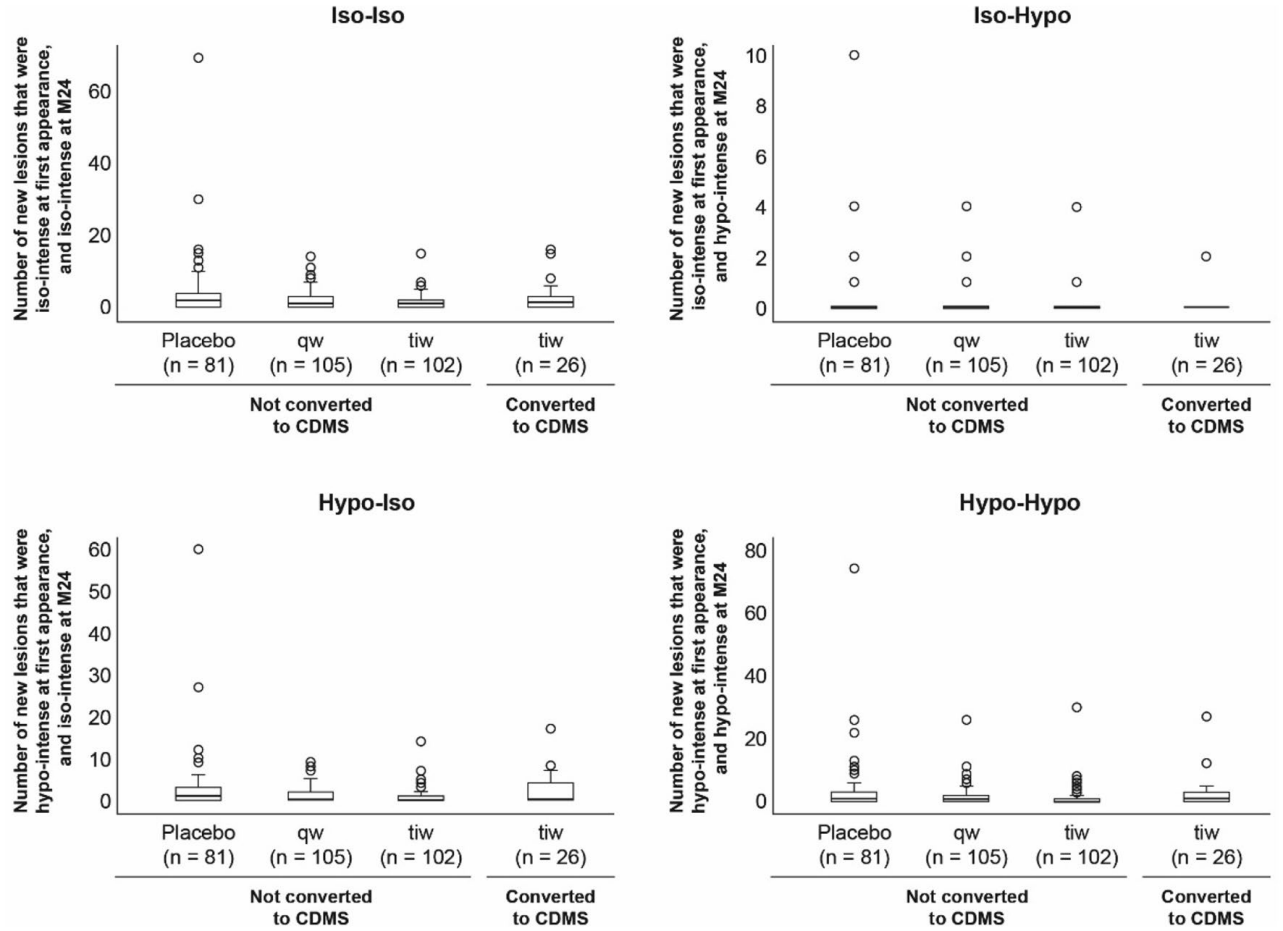
Box and whisker plots of evolution of new lesions during the 24-month study period in patients with a first clinical demyelinating event and treated with placebo or subcutaneous interferon β-1a (once- or three-times-weekly). Findings are shown according to whether patients converted to clinically definite multiple sclerosis or not. Lesions were classified into four categories based on their intensity at first appearance and at M24, reflecting the extent and development/persistence of demyelination and axonal loss. Iso–iso: number of new lesions that were iso-intense at first appearance and iso-intense at M24; Iso–hypo: number of new lesions that were iso-intense at first appearance and hypo-intense at M24; hypo–iso: number of new lesions that were hypo-intense at first appearance and iso-intense at M24; hypo–hypo: number of new lesions that were hypo-intense at first appearance and hypo-intense at M24. *CDMS* clinically definite multiple sclerosis, *M* month, *qw* once-weekly, *tiw* three-times-weekly

These analyses included only those patients in the sc IFN β-1a tiw treatment group (*n* = 128), of whom 26 patients converted to CDMS. The latter patients had more new lesions overall, more new lesions that were hypo-intense at M24, and more new lesions that were iso-intense at M24, compared with non-converters (Table 2, Fig. 1). Patients converting to CDMS also had higher numbers of iso–iso, hypo–iso, and hypo–hypo-lesions (Table 2, Fig. 2). Both the number of new lesions that were hypo-intense on the majority of scans and the number that were iso-intense were significantly elevated in patients converting to CDMS compared with those who did not convert (Table 2, Fig. 1).

**Table 2:** New lesions per patient identified over 24 months, according to treatment group

New lesion type [median (Q1, Q3) number]	Placebo	sc IFN β-1a 44 μg qw	sc IFN β-1a 44 μg tiw, *n* = 128	
	Non-converted to CDMS *n* = 81	Non-converted to CDMS *n* = 105	Non-converted to CDMS *n* = 102	Converted to CDMS *n* = 26
Any (overall)	4.0 (2.0, 10.0)	3.0 (1.0, 6.5)**	1.0 (0.0, 4.0)***^†^	3.5 (0.0, 11.0)*
M24-iso	3.0 (1.0, 7.0)	1.0 (0.0, 4.0)**	1.0 (0.0, 3.0)***^†^	2.5 (0.0, 7.0)*
M24-hypo	1.0 (0.0, 3.0)	1.0 (0.0, 2.0)	0.0 (0.0, 1.0)***^††^	1.0 (0.0, 3.0)*
Majority-iso	1.0 (0.0, 4.0)	1.0 (0.0, 3.0)	0.0 (0.0, 2.0)**^†^	2.0 (0.5, 4.0)*
Majority-hypo	4.0 (1.0, 7.0)	2.0 (1.0, 5.0)**	2.0 (1.0, 3.0)***	4.0 (2.0, 6.0)*
Iso–iso	2.0 (0.0, 4.0)	1.0 (0.0, 3.0)**	1.0 (0.0, 2.0)***	1.5 (0.0, 3.0)*
Iso–hypo	0.0 (0.0, 0.0)	0.0 (0.0, 0.0)	0.0 (0.0, 0.0)*	0.0 (0.0, 0.0)
Hypo–iso	1.0 (0.0, 3.0)	0.0 (0.0, 2.0)**	0.0 (0.0, 1.0)***^††^	0.0 (0.0, 4.0)*
Hypo–hypo	1.0 (0.0, 3.0)	1.0 (0.0, 2.0)	0.0 (0.0, 1.0)***^††^	1.0 (0.0, 3.0)*

Levels of significance (*P < 0.05; **P < 0.01; ***P < 0.001) refer to comparisons between non-converted to CDMS patients treated with sc IFN β-1a qw or tiw versus placebo or comparisons between patients treated with sc IFN β-1a tiw who converted to CDMS versus those who did not convert to CDMS (non-converted). Levels of significance (^†^*P* < 0.05; ^††^*P* < 0.01) refer to comparisons between patients who did not convert to CDMS who were treated with sc IFN β-1a tiw versus qw. The column “New lesion type” denotes the type of new lesion, as follows. Any (overall): overall new lesions per patient up to and including M24; M24-Iso: new lesions that were iso-intense at the M24 time point; M24-Hypo: new lesions that were hypo-intense at the M24 time point; Majority-Iso: new lesions that were iso-intense on the majority of available time points; Majority-Hypo: new lesions that were hypo-intense on the majority of available time points; Iso–iso: new lesions that were iso-intense at first appearance and iso-intense at M24; Iso–hypo: new lesions that were iso-intense at first appearance and hypo-intense at M24; Hypo–iso: new lesions that were hypo-intense at first appearance and iso-intense at M24; hypo–hypo: new lesions that were hypo-intense at first appearance and hypo-intense at M24

*CDMS* clinically definite multiple sclerosis, *FCDE* first clinical demyelinating event, *IFN* interferon, *M24* Month 24, *Q* quartile, *qw* once-weekly, *sc* subcutaneous, *tiw* three-times-weekly

#### Effects of treatment on black-hole lesions

These analyses included only those patients who did not convert to CDMS in both sc IFN β-1a groups and the placebo group (*n* = 290). Compared with patients treated with placebo, those treated with sc IFN β-1a tiw or qw had fewer new lesions overall, as well as fewer new lesions that were iso-intense at M24 (Table 2, Fig. 1). In addition, the number of new lesions that were hypo-intense at M24 was reduced in patients treated with sc IFN β-1a tiw, but not in those treated with sc IFN β-1a qw, compared with placebo recipients (Table 2, Fig. 1). Directly comparing the active treatment groups, those treated with sc IFN β-1a tiw had fewer new lesions overall, as well as fewer new lesions that were iso-intense or hypo-intense at M24 (Table 2, Fig. 1).

With respect to evolution of lesion intensities over time, the number of iso–iso and hypo–iso-lesions was significantly reduced in both active treatment groups, compared with placebo (Table 2, Fig. 2). The numbers of iso–hypo and hypo–hypo-lesions were reduced in patients treated with sc IFN β-1a tiw, but not in those treated with sc IFN β-1a qw, compared with in placebo-treated patients (Table 2, Fig. 2). In direct comparisons between the two active treatment groups, those treated with sc IFN β-1a tiw had reduced numbers of hypo–iso- and hypo–hypo-lesions versus sc IFN β-1a qw (Table 2, Fig. 2).

Finally, we analysed the appearance of new lesions at the majority of the available time points. Patients treated with either sc IFN β-1a regimen had fewer new lesions that were hypo-intense on the majority of scans than those treated with placebo (Table 2, Fig. 1). Furthermore, patients treated with sc IFN β-1a tiw, but not those treated with sc IFN β-1a qw, had fewer new lesions that were iso-intense on the majority of scans than those treated with placebo (Table 2, Fig. 2).

During analysis, we noticed that there was a large and unexpected outlier (Figs. 1 and 2). This patient was retained within the analysis following a visual inspection confirming the presence of new lesions. The analysis was repeated with this outlier excluded, and we were reassured to observe no change in the results.

### Central atrophy

MRI scans from 291 patients were available for analysis (sc IFN β-1a tiw, *n* = 116 [converters to CDMS, *n* = 25; non-converters, *n* = 91]; sc IFN β-1a qw, *n* = 98 [all non-converters]; placebo, *n* = 77 [all non-converters]; Supplementary Fig. 1). Baseline demographics and characteristics of the groups were generally similar (Table 1).

#### Central brain atrophy in non-converters

**Fig. 3:**
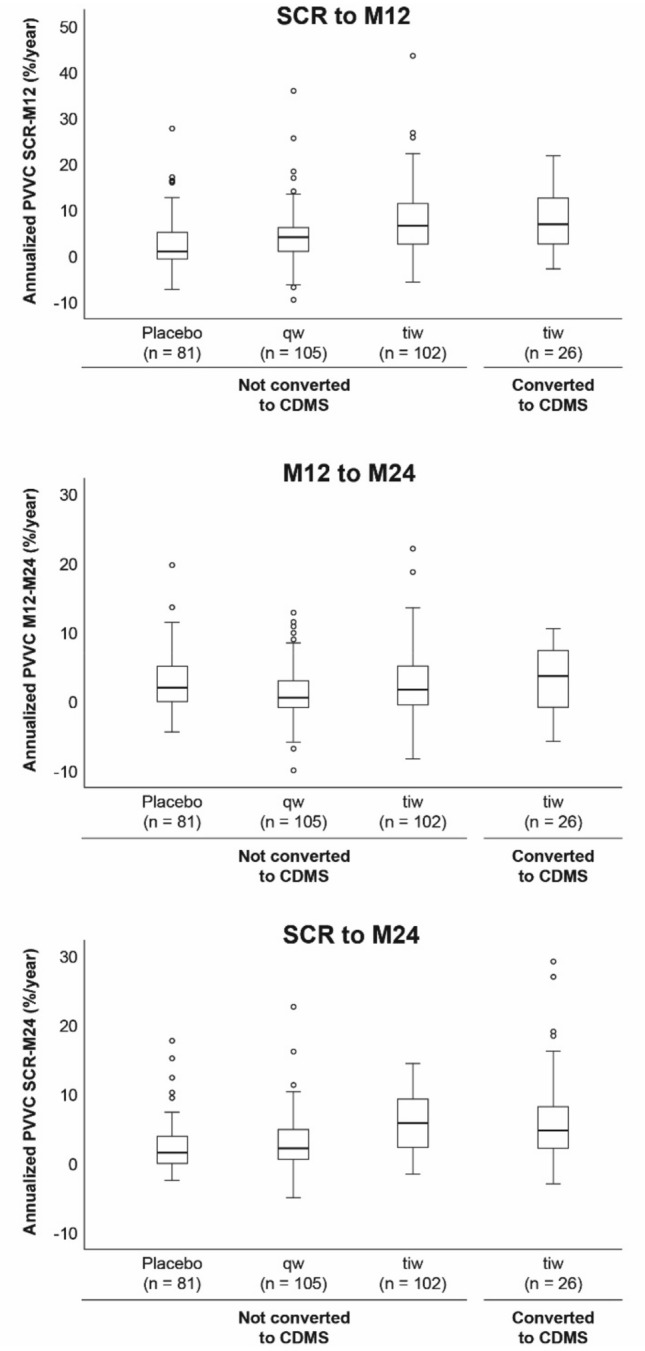
Box and whisker plots of annualized ventricular enlargement rates at different time intervals in patients with a first clinical demyelinating event and treated with placebo or subcutaneous interferon β-1a (once- or three-times-weekly). Findings are shown according to whether patients converted to clinically definite multiple sclerosis or not. *CDMS* clinically definite multiple sclerosis, *M* month, *PVVC* percentage ventricular volume change, *qw* once-weekly, *SCR* screening, *tiw* three-times-weekly

These analyses included only patients treated with sc IFN β-1a tiw (*n* = 114). Annualized PVVC did not differ between patients who converted to CDMS and those who did not convert for any of the three time intervals (screening–M12, M12–M24 and screening–M24; Table 3, Fig. 3).

**Table 3:** The effect of subcutaneous interferon β-1a (once- or three-times-weekly) versus placebo on annualized ventricular enlargement rates per patient with respect to conversion status to clinically definite multiple sclerosis, at different time intervals

Annualized PVVC over time, %/year [median (Q1, Q3)]	Placebo	sc IFN β-1a 44 μg qw	sc IFN β-1a 44 μg tiw, *n* = 116	
	Non-converted to CDMS *n* = 77	Non-converted to CDMS *n* = 98	Non-converted to CDMS *n* = 91	Converted to CDMS *n* = 25
SCR to M12	1.11 (− 0.47, 5.24)	4.28 (1.21, 6.29)**	6.76 (2.92, 11.76)***^‡^	6.92 (2.74, 12.68)
M12 to M24	2.27 (0.32, 5.33)	1.01 (− 0.71, 3.33)*	2.05 (− 0.19, 5.42)	3.94 (− 0.53, 7.62)
SCR to M24	1.51 (0.26, 3.88)	2.21 (0.57, 4.79)	4.71 (2.24, 7.92)***^‡^	5.84 (2.41, 9.31)

Levels of significance (*P < 0.05; **P < 0.01; ***P < 0.001) refer to comparisons between non-converted to CDMS patients treated with sc IFN β-1a qw or tiw versus placebo; no comparisons between patients treated with sc IFN β-1a tiw who converted to CDMS versus those who did not convert to CDMS (non-converted) were significant. Levels of significance (^‡^*P* < 0.001) refer to comparisons between patients who did not convert to CDMS who were treated with sc IFN β-1a tiw versus qw

*CDMS* clinically definite multiple sclerosis, *IFN* interferon, Mi12 Month 12, *M24* Month 24, *PVVC* percentage ventricular volume change, *qw* once-weekly, *sc* subcutaneous, *SCR* screening, *tiw* three-times-weekly

#### Onset of central brain atrophy with sc IFN β-1a versus placebo

These analyses included only those patients who did not convert to CDMS. Between screening–M12, patients treated with sc IFN β-1a tiw or qw had a higher annualized PVVC than those treated with placebo (Table 3, Fig. 3). Between M12–24, patients treated with sc IFN β-1a qw, but not those treated with sc IFN β-1a tiw, had a significantly reduced annualized PVVC compared with placebo recipients (Table 3, Fig. 3). Over the 2-year study period, annualized PVVC was higher in patients receiving sc IFN β-1a tiw versus placebo, whereas no difference was observed between patients receiving sc IFN β-1a qw and placebo (Table 3, Fig. 3).

## Discussion

MRI has emerged as a valuable tool for diagnosis, detection of subclinical disease activity, and monitoring disease progression/response to treatment in patients with MS [21]. In this regard, chronic black holes have been shown to correspond with regions of irreversible tissue damage and particularly with histopathological reductions in axonal density [7, 8, 10, 12]. Furthermore, increasing T1-hypo-intense lesion loads have been linked with increasing disability [22, 23]. Our findings appear to suggest that, by reducing the extent of axonal loss, treatment with sc IFN β-1a tiw may slow disease progression. Overall, 2 year treatment with sc IFN β-1a tiw, but not sc IFN β-1a qw, significantly reduced the development of black holes compared with placebo in patients who did not convert to CDMS. Specifically, fewer lesions evolving from iso- to hypo- and remaining hypo-intense were detected in patients treated with sc IFN β-1a tiw, while the number of new lesions that were hypo-intense at the majority of time points was also reduced. Additionally, treatment with sc IFN β-1a tiw improved all black-hole lesion parameters among patients who did not convert to CDMS.

As all black-hole numbers were reduced by treatment, especially in the tiw group, the number of initially hypo-intense lesions subsequently evolving to iso-intense lesions (the “hypo-iso” lesions) was also lower in that group. This should not be interpreted as a lack of ability to repair, but rather as successful prevention of new lesion formation. In patients treated with sc IFN β-1a tiw, those who converted to CDMS had significantly greater numbers of new lesions, including new hypo-intense lesions, than non-converters. However, while converters had higher numbers of hypo–hypo-lesions, there was no significant difference between converters and non-converters in the number of iso–hypo-lesions. It should be noted that due to the small sample size of the population treated with sc IFN β-1a tiw who converted to CDMS, this result should be treated with caution.

Previous analyses of patients in the REFLEX study showed that brain atrophy, measured by percentage brain volume loss in comparison with placebo, appeared to be greater with sc IFN β-1a tiw (and similar with sc IFN β-1a qw) at M24 [6]. This finding was partly attributed to pseudo-atrophy in the early treatment phase resulting from the anti-inflammatory actions of sc IFN β-1a. In line with these findings, the current analysis showed that ventricular enlargement in the first year of treatment with either sc IFN β-1a regimen was greater than for placebo; we similarly propose that this is likely due to pseudo-atrophy [24, 25]. In an analogous fashion, this likely pseudo-atrophy was greatest with sc IFN β-1a tiw, in agreement with the clinical findings that show a more pronounced effect on focal inflammatory activity of sc IFN β-1a with more frequent dosing [5]. From Month 12–24, in patients who did not convert to CDMS, the annualized PVVC rate with sc IFN β-1a tiw was similar compared to placebo, and reduced with sc IFN β-1a qw, suggesting that the effect of pseudo-atrophy may have resolved.

In patients treated with sc IFN β-1a tiw, those who converted to CDMS did not have faster ventricular enlargement (annualized PVVC) than non-converters. There is evidence in the literature that early central atrophy is predictive, as well as a correlate of poorer prognosis in patients with relapse-onset MS and minimal impairment, with similar, but non-significant, findings in the small subset of patients with FCDE [26]. It is likely that the small size of the group of patients who converted to CDMS in spite of sc IFN β-1a treatment in this study hampered detection of differences. Indeed, to compare findings in patients who converted to CDMS with those in patients who did not convert, the analysis group had to be restricted to only those patients who were treated with sc IFN β-1a tiw; consequently, the group size for analysis was limited.

### Limitations

Interpretation of the present analysis is complicated by the time-to-event design of the REFLEX study. Among converters to CDMS, only patients from the original sc IFN β-1a tiw treatment group received the same treatment throughout the study. Therefore, patients initially treated with placebo or sc IFN β-1a qw were excluded to give a homogeneous population of converters to CDMS. This resulted in a low number of patients for analysis, reducing the power to detect any differences between converter and non-converter groups. In addition, this approach may have introduced a small bias: by retaining CDMS converters in the tiw group but not the other groups, the patients in the tiw group could appear to be doing less well. However, in the relevant analyses, the converters and non-converters were separated into two different groups, allowing a fair comparison to the tiw non-converters only. Another limitation of this study was the poor contrast between GM and WM in 2D T1-weighted images, which prevented us from performing an assessment of tissue atrophy. To make the analysis more sensitive, we limited the atrophy assessment to the ventricular regions using VIENA, a tool specifically designed for use in images with poor contrast. Interpretation of our findings may also be limited by is the fact that we used a binary classification (presence or absence) of black-hole status based on visual inspections and, as such, these black-hole measures are not quantitative by nature [27]. Further investigation, perhaps using more advanced quantitative imaging techniques [28], are therefore warranted, along with an analysis of the interdependence of the association between conversion to CDMS, T1 hypo-intense lesion formation, and cumulative treatment exposure. Finally, in view of the retrospective nature of the analysis, reported p values were descriptive.

### Conclusions and future directions

Compared with placebo, treatment with sc IFN β-1a tiw, but not sc IFN β-1a qw, reduced the evolution of the absolute number of new lesions into black holes in patients with a FCDE and was associated with an increase of the annualized PVVC. Given the strong correlation between the presence of T1 hypo-intensity (black holes) and reduced axonal density [29], these findings are of clinical relevance to patients with MS. An area of future interest is whether lesions that develop during treatment differ from those that develop in untreated patients.

## Supplementary Information

Below is the link to the electronic supplementary material.

Supplementary file1 (DOCX 50 KB)

## Data Availability

Any requests for data by qualified scientific and medical researchers for legitimate research purposes will be subject to Merck’s Data Sharing Policy. All requests should be submitted in writing to Merck’s data sharing portal https://www.merckgroup.com/en/research/our-approach-to-research-and-development/healthcare/clinicaltrials/commitment-responsible-data-sharing.html. When Merck has a co-research, co-development, or co-marketing or co-promotion agreement, or when the product has been out-licensed, the responsibility for disclosure might be dependent on the agreement between parties. Under these circumstances, Merck will endeavour to gain agreement to share data in response to requests.
