# Supplementary material for: Temporal evolution of new T1-weighted hypo-intense lesions and central brain atrophy in patients with a first clinical demyelinating event treated with subcutaneous interferon β-1a
Source: J Neurol. 2023 Feb 1;270(4):2271–82. doi: 10.1007/s00415-022-11554-5 (PMC10025187; doi:10.1007/s00415-022-11554-5)
Supplement: Supplementary file 1 — Supplementary file1 (DOCX 50 KB) [file 415_2022_11554_MOESM1_ESM.docx]

**Supplementary Figure 1:** Patient disposition
CDMS, clinically definite multiple sclerosis; MRI, magnetic resonance imaging; qw, once-weekly; sc IFN β-1a, subcutaneous interferon β-1a; tiw, three-times-weekly.

**Analysis population**

**Original trial, n = 458**
Placebo, n = 154

sc IFN β-1a 44 μg qw, n = 152

sc IFN β-1a 44 μg tiw, n = 152

**Patients with scans available for *post hoc* MRI analyses, n = 410**

Placebo, n = 138

sc IFN β-1a 44 μg qw, n = 138

sc IFN β-1a 44 μg tiw, n = 134

**No MRI scan available at Month 12 and/or <2 scans available after Month 12, n = 48**

Placebo, n = 16

sc IFN β-1a 44 μg qw, n = 14

sc IFN β-1a 44 μg tiw, n = 18

**Initial lesion evolution analyses population, n = 388**

Placebo, n = 128

sc IFN β-1a 44 μg qw, n = 132

sc IFN β-1a 44 μg tiw, n = 128

**Image and/or lesion mask or brain mask incorrect, incomplete, or incompatible,
n = 22**

Placebo, n = 10

sc IFN β-1a 44 μg qw, n = 6

sc IFN β-1a 44 μg tiw, n = 6

**Converted to CDMS and changed treatment, n = 74**

Placebo, n = 47

sc IFN β-1a 44 μg qw, n = 27

sc IFN β-1a 44 μg tiw, n = 0

**Analysis pipeline failure, n = 33**

Placebo, n = 4

sc IFN β-1a 44 μg qw, n = 7

sc IFN β-1a 44 μg tiw, n = 12

**Final lesion evolution analyses population,
n = 314**

Placebo, n = 81

sc IFN β-1a 44 μg qw, n = 105

sc IFN β-1a 44 μg tiw, n = 128

Converters to CDMS, n = 26

Non-converters to CDMS, n = 102

**Central atrophy analyses population,**

**n = 291**

Placebo, n = 77

sc IFN β-1a 44 μg qw, n = 98

sc IFN β-1a 44 μg tiw, n = 116

Converters to CDMS, n = 25

Non-converters to CDMS, n = 91
